# Multimodal phenotypic clustering predicts cardiac outcomes in Fabry disease

**DOI:** 10.1186/s13023-026-04401-7

**Published:** 2026-06-17

**Authors:** Elad Shemesh, Paul Feigin, Chong Yew Tan, Rosemary Rusk, Deepa Gopalan, Patrick Deegan

**Affiliations:** 1 Population Health Research Institute, David Braley Cardiac, Vascular and Stroke Research Institute, Hamilton Health Sciences and McMaster University, Hamilton, Canada; 2 Technion- Israel Institute of Technology, Haifa, Israel; 3 Lysosomal Disorders Unit, Cambridge University Hospitals NHS Trust, Cambridge, UK; 4 Department of Cardiology, Cambridge University Hospitals NHS Trust, Cambridge, UK; 5 Department of Radiology, Cambridge University Hospitals NHS Trust, Cambridge, UK; 6 Department of Radiology, Imperial College Healthcare NHS Trust, London, UK; 7 Clinical Research Laboratory & Biobank Genetic & Molecular Epidemiology Laboratory (CRLB-GMEL), 237 Barton Street East, Hamilton, ON L8L2X2 Canada

**Keywords:** Fabry, Cardiomyopathy, Heart failure

## Abstract

**Background:**

Fabry disease (FD) is a rare lysosomal storage disorder with cardiac involvement. The efficacy of cardiac assessments in predicting disease progression is uncertain and few long-term studies have evaluated a clinically comprehensive approach in tracking cardiac outcomes. We developed a multi-modal framework integrating sex, genetics, clinical assessments, electrocardiography (ECG), imaging, and biomarkers to characterize the progression of cardiac involvement in FD and assess the predictive value of each modality for adverse cardiovascular outcomes.

**Results:**

Our cohort encompassed 525 “visit years” across 98 participants (60 females; 60 with classic mutations; mean age at presentation: 42.5 ± 17). We defined four cardiac phenotypic clusters (CPC): abnormal ECG, elevated cardiac biomarkers, left ventricular hypertrophy (LVH) (by echocardiography or cardiac magnetic resonance [CMR]), and late gadolinium enhancement (LGE) (by CMR). The primary outcome was a composite of cardiac hospitalization, device implantation, or cardiovascular/cerebrovascular death. A CPC progression map was plotted, outlining the course of events in FD cardiomyopathy, where changes in ECG and the occurrence of hypertrophy precede increase in cardiac biomarkers and the formation of LGE. Mutation type (classic vs. late-onset) and sex (female vs. male) showed a significant link with a composite outcome of cardiac-related hospitalizations, device implantations (defibrillator/pacemaker), and cardiovascular or cerebrovascular mortality [Hazard ratio, HR (95% confidence interval, CI, p-value): 12.8 (3.5,46.7), *p* = 0.0001 and 0.09 (0.03,0.33), *p* = 0.0002, respectively]. The presence of LGE CPC was associated with an increased likelihood of encountering the composite outcome.

**Conclusions:**

We propose a readily-applied “4 domain CPC” model, that can be routinely used in clinical practice, as a prospective “guide-map” for FD cardiomyopathy progression. Its utility is demonstrated in showing the expected role of sex, mutation and CMR findings in the cardiac trajectory of FD.

**Supplementary Information:**

The online version contains supplementary material available at https://doi.org/10.1186/s13023-026-04401-7.

## Introduction

Fabry disease (FD) (OMIM 30150) is a rare X-linked lysosomal storage disorder caused by mutations in the *GLA* gene, which encodes the enzyme α-galactosidase A [1]. Deficiency or absence of this enzyme leads to the accumulation of globotriaosylceramide (Gb3) in lysosomes of multiple organs, notably the heart [2]. The birth prevalence of FD has been estimated at 1:40,000–170,000 [3], although newborn screening suggests the true incidence may be higher [4]. Patients often experience diagnostic delays exceeding a decade, and by the time FD is confirmed, cardiac involvement—in the form of ventricular hypertrophy, myocardial fibrosis, arrhythmias, conduction disturbances, and heart failure—may already be present or progress rapidly [5, 6].

Two main phenotypes are recognized: the “classic” form, which typically manifests in males with neuropathic pain, angiokeratomas, dysmorphic facial features, and multisystem organ dysfunction; and a “late-onset” form that predominantly affects the heart [7]. In females heterozygous for FD, lyonization (X-chromosome inactivation) leads to highly variable clinical presentations, as disease severity depends on the balance of inactivation of the mutant or wild-type allele throughout the cells of the body [8].

A range of modalities is available to assess cardiac involvement in FD. Electrocardiography (ECG) can detect bradycardia, arrhythmias, atrio-ventricular block, and repolarization abnormalities, though the prognostic value of these findings remains unproven [6]. Cardiac biomarkers, such as troponin and NT-proBNP, are frequently elevated, but may be influenced by the concurrent renal dysfunction that is common in FD [9]. Imaging techniques, particularly echocardiography and cardiac magnetic resonance (CMR), are central to evaluating left ventricular hypertrophy, mass, and function. In addition, CMR can detect late gadolinium enhancement (LGE) indicative of myocardial fibrosis, which may hold prognostic significance [10, 11]. Nevertheless, echocardiography depends on normal cardiac geometry assumptions—potentially limiting accuracy in FD—while CMR, though highly informative, is costlier and less accessible in many regions.

Despite the recognized utility of these individual tools, current clinical practice rarely synthesizes genetics, clinical baseline and outcome data, ECG, biomarker, and imaging findings into a unified predictive model for FD cardiomyopathy. Nor is there clarity on the optimal sequencing or frequency of these tests to best anticipate disease progression. As a result, the natural history of cardiac involvement in FD remains incompletely defined, and the capacity to forecast patients’ disease trajectories—particularly in a heterogeneous condition influenced by sex, genotype, and phenotype—remains limited.

In this study, we propose an integrated, multi-modal framework that combines genotypic data, serial imaging, cardiac biomarkers, ECG findings, and clinical outcomes to construct predictive trajectories for FD-related cardiac disease. By reframing existing diagnostic methods into a cohesive tool, we aim to refine risk stratification and inform personalized monitoring strategies—ultimately improving our understanding and management of FD cardiomyopathy.

## Methods

### Study population

Subjects with FD who had undergone at least two cardiac evaluations at Cambridge University Hospitals between 2003 and 2019 were recruited. 112 subjects were screened for inclusion. Five subjects were not confirmed to have FD, and three had insufficient clinical data recordings. Of the remaining 104 subjects, 6 subjects had no measurements before the cut-off date (2019-06-30) as well as no recorded endpoint (for death, device implantation, or hospitalization). In total, 98 subjects met the inclusion criteria, corresponding to 703 patient years.

In England, there are five designated centres for the management of adult patients with FD. Patients cannot receive state-funded specific treatment for FD unless they attend one of the designated centres. Patients may be referred because of a new diagnosis (often as a result of the application of genetic panels in patients attending cardiology clinics presenting with unexplained left ventricular hypertrophy) or as a result of testing of at-risk family members of index patients. Our hospital is one of the five adult reference centres. The study received Research Ethic Committee approval (IRAS ID 265545). Ethical review was conducted by a Research Ethics Committee (REC) within the UK Health Departments’ Research Ethics Service (NHS/HSC RECs). As patients did not undergo any study procedures and as only aggregate data is reported, it was adjudicated that individual informed consent was not required. As a result of this, individual genetic data cannot be reported as part of this study.

### Genotype classification

A patient’s genotype was classified into ‘classic’ or ‘late-onset’ based on the Fabry-Gen-Phen: The Fabry Working Group Genotype Phenotype Database (http://fabrygenphen.com). Female subjects were classified according to genotype rather than phenotype, in other words the affected female relative of a male with a classic genotype was classified as “classic” regardless of her own disease phenotype [12, 13]. This is in contrast to studies where female patients are classified according to their own clinical phenotype at any given time [14].

### Cardiac evaluations

As part of evolving clinical practice, cardiac examinations of FD patients included ECG, blood testing for cardiac biomarkers, echocardiographic studies and CMR. The availability of these varied over time due to technical advancements, such as the digitalization of ECG images in our hospital in 2014 and the increased feasibility of CMR in the last decade. The methodology of measuring cardiac biomarkers (i.e. troponin and high sensitivity troponin) changed during the study. To address the challenges that arise in maintaining uniformity of data collection in the face of changing methods and evolving medical practice, we focused on key variables consistently reported for each modality using a dichotomous approach. These variables included: (1) ECG: Rate, rhythm, PR interval, QRS width, T wave inversion; (2) Echocardiography: Left ventricular hypertrophy (LVH) measurement locations and cut-offs were based on the American Heart Association (AHA) guideline for diagnosis of hypertrophic cardiomyopathy, based on maximal end-diastolic wall thickness evaluations greater than 15 mm. Septal measurement greater than 13 mm in males, or greater than 11 mm for females, was also considered as an additional criteria for definition of hypertrophy in this known, genetic-proven, population of FD patients; (3) CMR: Left ventricular hypertrophy (LVH), LGE; (4) Biomarkers: blood concentrations of Troponin and NT-proBNP based upon a threshold of the upper reference limit for individual assays.

### Adverse cardiovascular outcomes

Adverse cardiovascular outcomes were defined as the occurrence of either cardiac-related hospitalizations, an implantation of a defibrillator and/or pacemaker, a cardiovascular disease-related (CVD) and/or cerebrovascular death. The primary endpoint was a composite of the above outcomes, reached when the patient first met any of the nominated adverse cardiovascular conditions. Events were adjudicated by two physicians (one metabolic specialist and one cardiologist) by review of physical and electronic patient records.

### Definition of cardiac phenotypic clusters

For each patient and at any time point, Cardiac phenotypic clusters (CPC) were defined based on established reference intervals in 4 domains. We applied an ‘unbiased’ approach in our CPC analyses where any abnormality in a relevant measurement determined the CPC. In all cases, CPC = 0 indicated a measurement that was within the reference interval. Within the ECG domain, CPC 1 was defined as abnormal PR interval and/or QRS width, and/or documented T wave inversion. If a device had already been implanted and actively pacing, the ECG data was ignored, due to potential biases in interpreting ECGs when a pacemaker is in place. Within the hypertrophy domain, two investigational modalities contributed (Echocardiography and CMR). CPC 1 was defined based on echocardiographic thresholds followed the American Society of Echocardiography and the European Association of Cardiovascular Imaging recommendations for cardiac chamber quantification in adults [15]. CMR tests reporting LVH were also considered for the definition of this CPC, and in cases where discrepancy in the diagnosis of hypertrophy between CMR and echocardiography existed, CMR took precedence. Within the LGE domain, CPC 1 was defined as presence of LGE in CMR. Within the Biomarkers domain, CPC 1 was defined as either abnormally elevated troponin or NT-proBNP. In summary, for our analyses we considered 4 Domains, representing Hypertrophy, ECG, Biomarkers and LGE and in each domain CPC 0 represented values considered normal, and CPC 1 represented values considered abnormal.

Sankey plots were used to visualize patient flow across CPC states over time. Each node represents a distinct CPC combination, and the width of each connecting flow is proportional to the number of patients transitioning between states. Patients who reached the composite endpoint are represented as a terminal state.

### Data pre-processing

Given the “real-world” nature of the dataset and the complexity of following patients diagnosed with a rare disorder, visits were not always at regular intervals, and the measurement modalities used (Biomarker, Echo, ECG, CMR) varied between visits. For the 98 patients, the observed CPC data thus consisted of 1 to 4 CPC values at each visit for a given patient. The timescale used to analyze the data was the patients’ age (in whole years). “Visit years” were defined as the total number of patient-years in which at least one cardiac evaluation was recorded. Multiple visits that provided data for the same age of a given patient were combined – with the maximal CPC chosen if the same domain CPC was determined more than once. In survival analyses the CPC was assumed to remain at 1 after the first age at which it was so observed, although a few reversions from CPC 1 to CPC 0 did occur (Biomarkers – 6 patients, Hypertrophy- 8 patients, ECG- 3 patients) (supplementary Fig. 1). For modeling the effect of CPC on the time to endpoint, an initial CPC 0 (arbitrarily set at age 16, given the lack of ‘in-house’ data prior to this age and considering the infrequent observation of abnormalities at younger ages [6]) for each domain was assumed.

If none of those events was recorded before 30-06-2019, then the endpoint was censored at the age of the last recorded visit.

### Statistical analyses

The baseline characteristics of study participants were described as count (proportion) for categorical variables, and continuous variables were presented as mean (standard deviation). These characteristics were computed separately for males and females (Table 1). The main descriptive analyses were based on comparative Kaplan-Meier survival curves for each CPC domain and for each endpoint. These were then compared for each gender-by-mutation (classic or late-onset) combination. For each CPC domain, survival curves were computed accounting for three censoring scenarios: (I) if CPC 1 was not observed during follow-up, the time was right-censored at the last assessment date for that modality; (II) if CPC 1 was present at the first available assessment, the time was treated as left-censored, as onset may have preceded observation; (III) if CPC 1 was first detected after the initial assessment, the event time was treated as uncensored. Patients for whom a given modality was never assessed did not contribute to the survival curve for that domain.

**Table 1:** Baseline (at first determination) characteristics of study cohort

	Males	ֿFemales
Age (years), mean ± SD	46.8 ± 16.3	39.7 ± 16.6
Mutation type, No. (%)		
Classic	20 (52.6%)	40 (67%)
Late-onset	18 (47.4%)	20 (33%)
Pathological ECG-DS, No. (%)*	23 (73.3%)	27 (51.9%)
Pathological Hypertrophy-DS, No. (%)*	27 (71%)	14 (23%)
Pathological biomarker-DS, No. (%)*	16 (44.%)	13 (22%)
Pathological LGE-DS, No. (%)*	14 (54%)	7 (15%)

ECG, Electrocradiopgrahy; DS, Disease state; LGE, Late gadolinium enhancement; SD, Standard deviation

* % among those who had at least one DS determination

In order to obtain an estimation of the predictive capability of the evolving CPC for the composite endpoint using the (evolving) CPC, Cox proportional hazards regression with time-dependent covariates was used. All Statistical analyses were performed in R version 4.2.3.

## Results

### Baseline characteristics

A total of 98 participants (38 males, 60 females) were included in this analysis. Of note, 60 subjects had a classic mutation. Table 1 depicts the baseline characteristics of the participants. The total number of “visit years” (defined above in terms of years of age) at which at least one modality was measured was 525. The number of observations for each CPC domain was: ECG- 211, Biomarkers- 322, Hypertrophy- 394, LGE- 147. Mean age at first visit was 42.5 ± 17. 60% (60%) had an abnormal ECG at first recording, 30% of patients had elevated cardiac biomarkers at first documented blood withdrawal (defined as elevated troponin and/or NT-proBNP), 42% had hypertrophy present, and 21% had LGE in CMR at first scan. Sixteen (16%) of the FD patients required implantation of a pacemaker and/or implantable cardioverter defibrillator. Throughout follow-up, 10 CVD-related hospitalizations (including 9 at age of death) were recorded. Composite events (cardiac-related hospitalizations, device implantations (defibrillator/pacemaker), and cardiovascular or cerebrovascular mortality) were recorded in 20 patients, a mean time-to-event of 7.5 years from first visit. (Table 2).

**Table 2:** Cardiac outcomes

	Males (*n* = 38)		Females (*n* = 60)	
	Classic (*n* = 20)	Late-onset (*n* = 18)	Classic (*n* = 40)	Late-onset (*n* = 20)
Cardiac hospitalizations and/or cardiovascular Death, No.	5	5	2	0
Device^~^ implantation, No.	7	3	5	1
Cardiac related death, No.	5	2	2	0
Composite events^#^, No.	8	5	6	1

HF, Heart failure

^~^Defined as either implantable cardioverter-defibrillator and/or pacemaker implantations

^#^Defined as a cardiac hospitalization, pacemaker and/or ICD implantations and cardiac-related and/or cerebrovascular death events

### The natural history of cardiac Fabry disease in this cohort

**Fig. 1:**
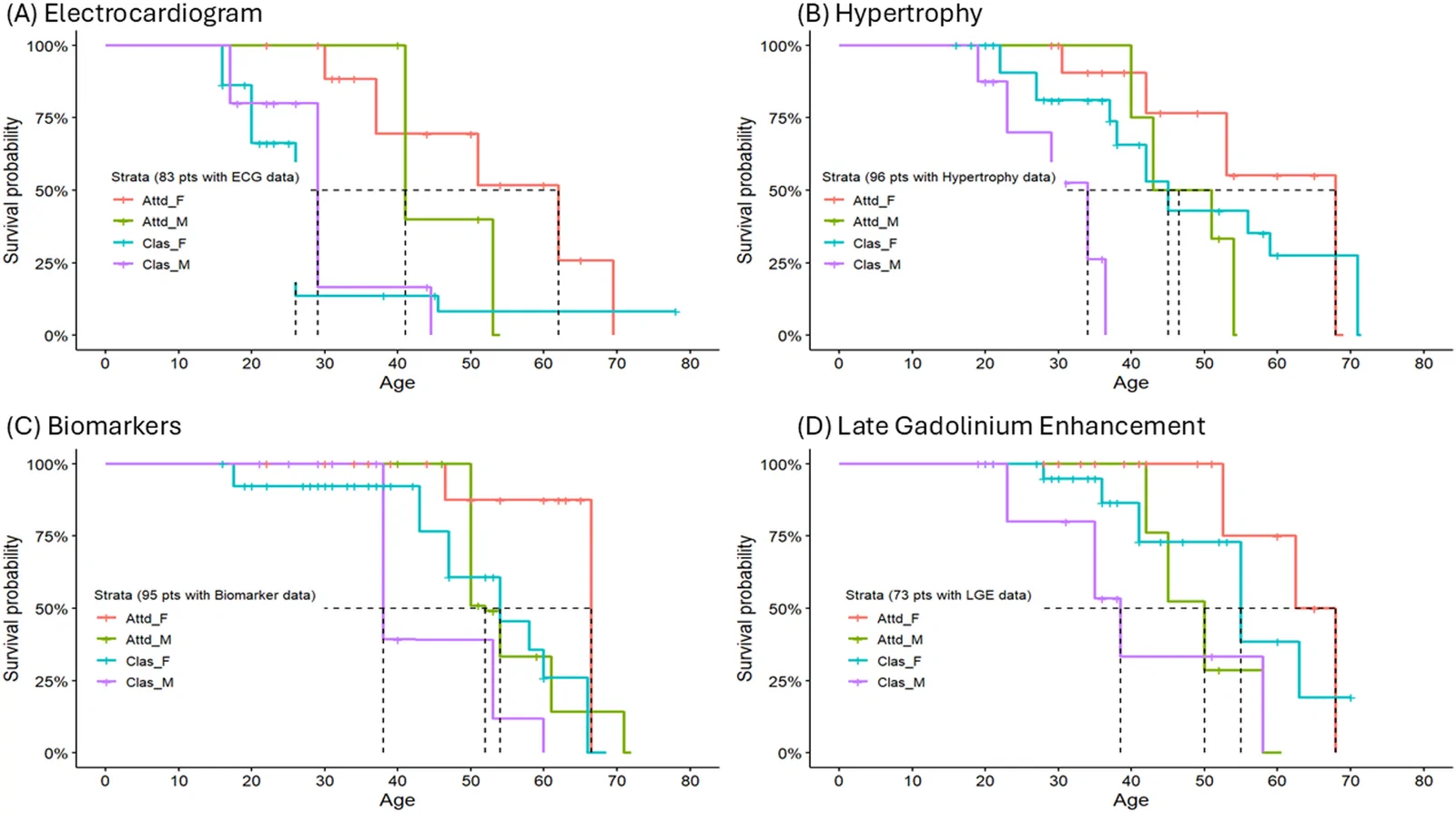
Survival curves for each Pathology (reaching CPC 1), stratified by mutation and sex. These curves take into account the censoring of the data: (**I**) if the pathology was not detected during follow-up, then the time assigned is the last time that pathology was assessed and it is marked as *right-censored*; (**II**) if the pathology was detected at the first time it was assessed, then that time is considered *left-censored* since the onset would have already occurred; (**III**) if the onset was detected after the first assessment, then the time of detection was treated as *un-censored*; (**IV**) if the particular pathology was never assessed for a given patient, then that patient does not contribute to the computation of the survival curve for that pathology

Figure 1 presents the survival curves for each CPC domain (ECG, biomarkers, hypertrophy, LGE), stratified by mutation and sex. Distinct trajectories are noted for the combination of sex and mutation: Generally, males carrying a classic mutation developed CPC 1 faster than their female peers, across all four domains. Females with a late-onset genotype were the least likely to develop CPC 1 throughout the follow-up time.

The estimated median age of onset of CPC 1 for each CPC domains stratified by mutation and sex is presented in Table 3. The median age of CPC 1 onset is at a later age for late-onset females in each cardiac domain, and at an earlier age for males carrying a classic mutation in most domains (Hypertrophy, LGE, Biomarkers). For the ECG domain, the median age of onset of CPC 1 was before 30 years of age for FD patients with classic mutation, whereas for those with a late-onset mutation, the median age of onset was of the order of 41 years for males, and 62 years for females. One must recall that data is not available for determining ECG pathologies after device implantation.

**Table 3:** Median age of detection of DS 1 across the different cardiac domains

Median age of detection	ECG	Hypertrophy	Biomarker	LGE	Composite Endpoint^#^	Hospital/Death Endpoint*
Late-onset Females	62	68	66.5	68	68	NA
Late-onset Males	41	49.5	52	50	73	71
Classic Females	26	45	54	55	74	83
Classic Males	29	34	38	38.5	51	59

ECG, Electrocardiogram; LGE, Late gadolinium enhancement

^#^Defined as a cardiac hospitalization, pacemaker and/or ICD implantations and cardiac-related and/or cerebrovascular death events

*Hospital/death endpoint does not take into account hospitalizations for ICD/pacemaker implantation

### Progression trajectories towards clinical events

Cox proportional hazards models were next used to investigate which, if any, of the CPC domains may be a predictor of reaching the endpoints of either heart failure hospitalization and/or cardiac-related death, or a composite of events that included device implantations. Beyond the significant effects of mutation and sex, LGE and ECG, to some extent, were also found as potential indicators of increased risk for reaching these outcomes. Of note, the addition of LGE to classic mutation increased the HR for cardiac hospitalizations and cardiac-related death events to 25.23 (P-value = 0.005), and to 16.53 (P-value = 0.0001) for a composite outcome that included device implantations. LGE was the only CPC that remained statistically significant when combined with mutation type and sex in predicting the composite outcome [HR-4.16, P-Value = 0.02]. Hypertrophy CPC added to mutation type and sex also resulted in statistically significant increase in risk for reaching the joint outcome of cardiac hospitalizations and/or cardiac-related death [HR-11.14, P-value = 0.03], but not for the composite of events including device implantations [HR-2.03, P-value = 0.19] (Table 4).

**Table 4:** The extra effect of disease state domains, beyond mutation and sex on composite cardiac outcomes

Third effect	Effect	Hazard ratio [95% CI]– cardiac hospitalizations and/or cardiac-related death^*^	*P*-Value	Hazard ratio [95% CI]– composite cardiac outcomes^#^	*P*-Value
None	Classic	12.87 [2.1-78.68]	**0.006**	12.79 [3.5-46.72]	**0.0001**
	Female	0.03 [0-0.31]	**0.003**	0.09 [0.03–0.33]	**0.0002**
Device Intervention	Classic	18.79 [4.45–79.31]	**0.0001**	NA	NA
	Female	0.44 [0.05–3.83]	0.46	NA	NA
	Interaction	0.15 [0.01–1.97]	0.15	NA	NA
ECG	Classic	8.96 [1.3-61.63]	**0.03**	11.07 [2.98–41.2]	**0.0003**
	Female	0.04 [0-0.38]	**0.005**	0.09 [0.03–0.32]	**0.0002**
	ECG	2.14 [0.48–9.46]	0.32	2.58 [0.9–7.38]	0.08
Hypertrophy	Classic	4.49 [0.57–35.61]	0.15	10.44 [2.77–39.33]	**0.0005**
	Female	0.12 [0.01–1.15]	0.07	0.12 [0.03–0.44]	**0.0001**
	Hypertrophy	11.14 [1.2-103.72]	**0.03**	2.03 [0.7–5.86]	0.19
Biomarkers	Classic	11.36 [1.68–76.85]	**0.01**	12.84 [3.37–48.98]	**0.0002**
	Female	0.04 [0-0.37]	**0.005**	0.09 [0.03–0.34]	**0.0003**
	Biomarkers	2.15 [0.48–9.75]	0.32	0.99 [0.28–3.47]	0.99
LGE	Classic	25.23 [2.66-239.15]	**0.005**	16.53 [4.17–65.52]	**0.0001**
	Female	0.02 [0-0.25]	**0.002**	0.09 [0.02–0.31]	**0.0002**
	LGE	6.34 [0.96–41.71]	0.055	4.16 [1.3-13.37]	**0.02**

ECG- electrocardiogram; LGE- Late Gadolinium Enhancement

* Hospital/death endpoint does not take into account hospitalizations for ICD/pacemaker implantation

#Defined as a cardiac hospitalization, pacemaker and/or ICD implantations and cardiac-related and/or cerebrovascular death events

**Fig. 2:**
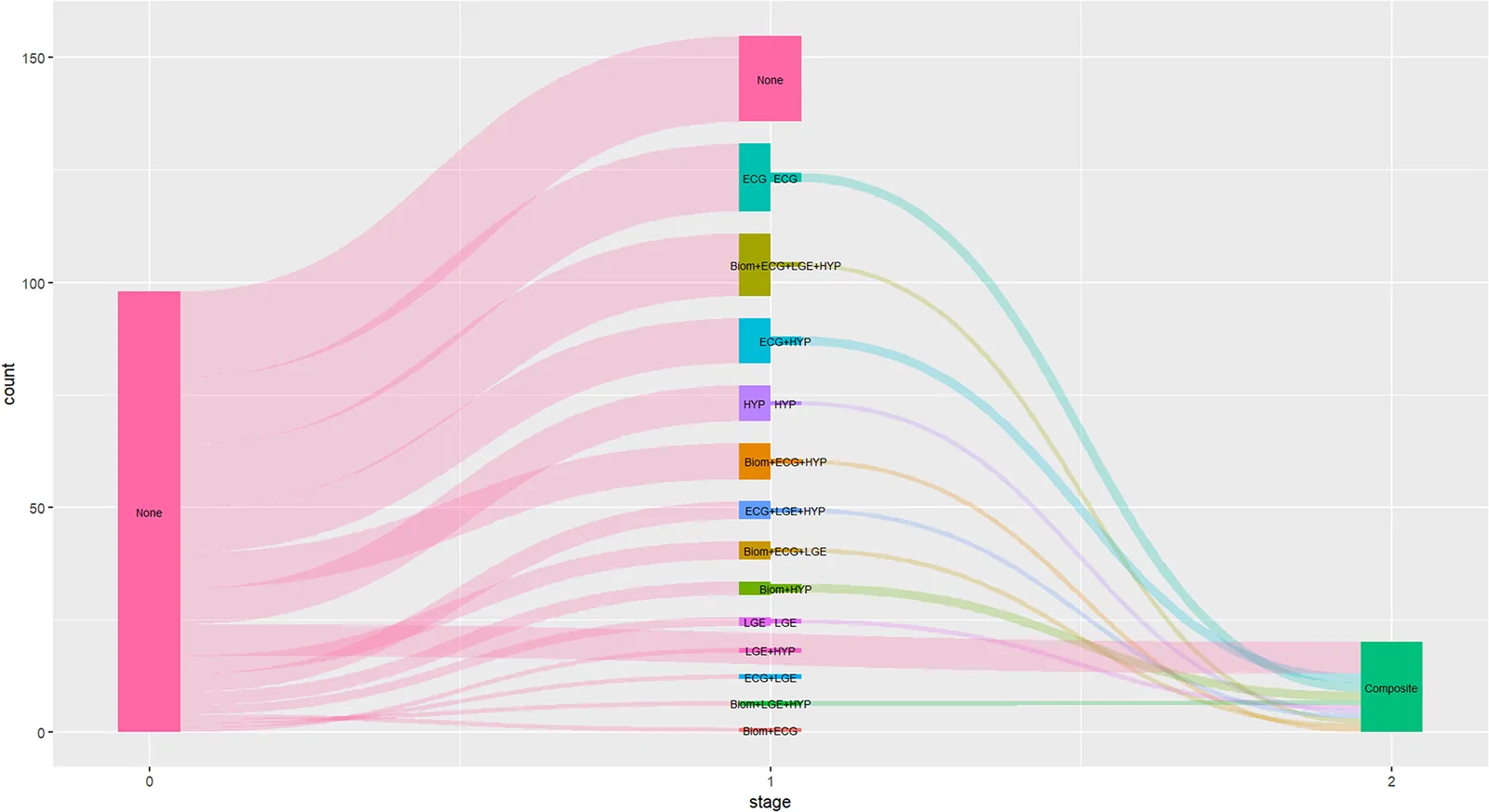
Sankey plot diagram for stage-based progression

**Fig. 3:**
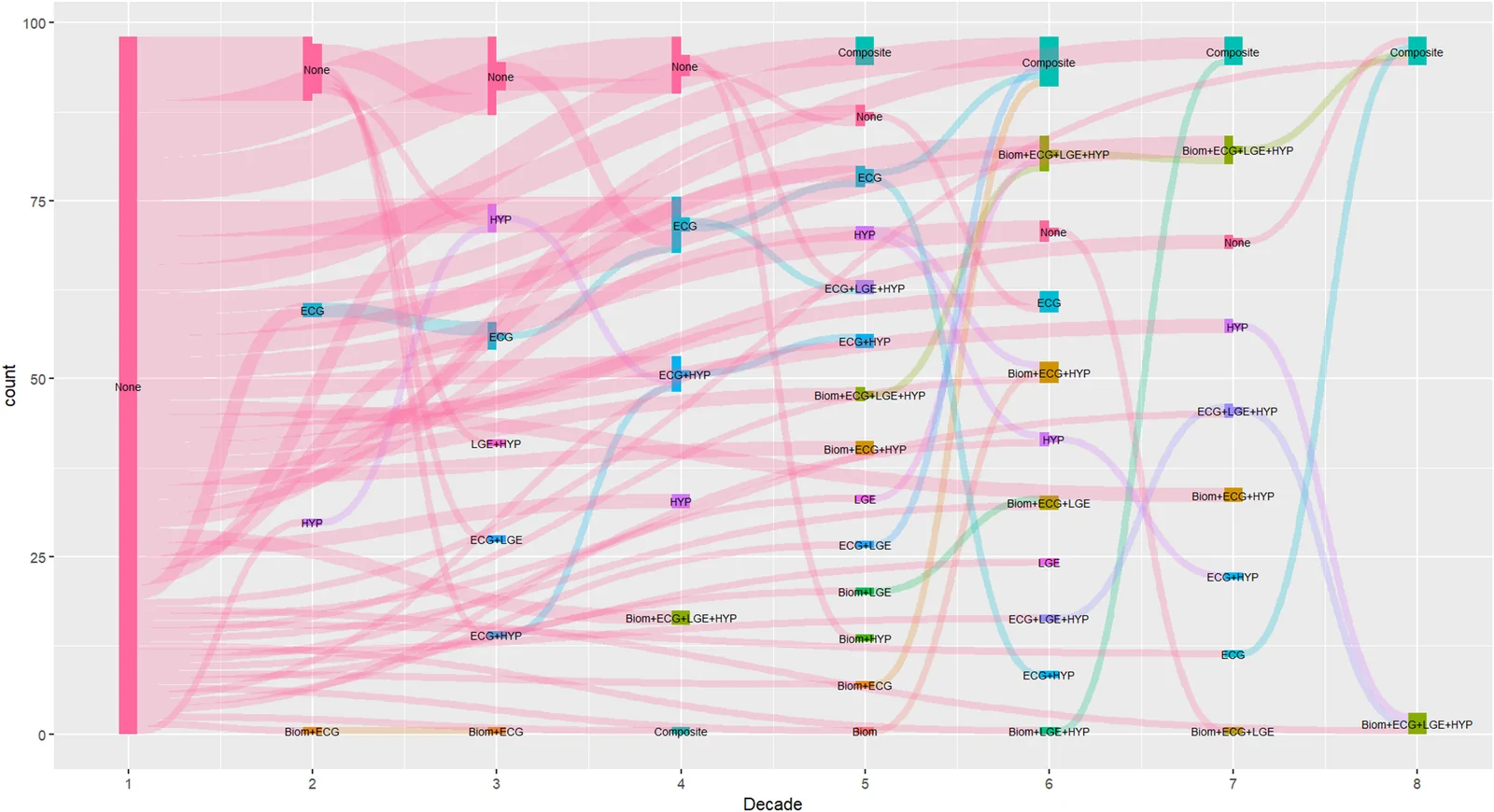
Sankey plot diagram for the cardiac progression of FD across CPCs over decades of life

Figure 2, a Sankey plot, visualizes how FD patients progress through stages of CPC. Figure 3 represents the longitudinal progression of CPC in FD across decades of life. Notably, most patients first transition to an abnormal ECG CPC, either alone or in combination with other abnormalities, before proceeding to more advanced CPC. A smaller subset of patients bypass these intermediate steps entirely and move directly into the composite outcome state.

**Fig. 4:**
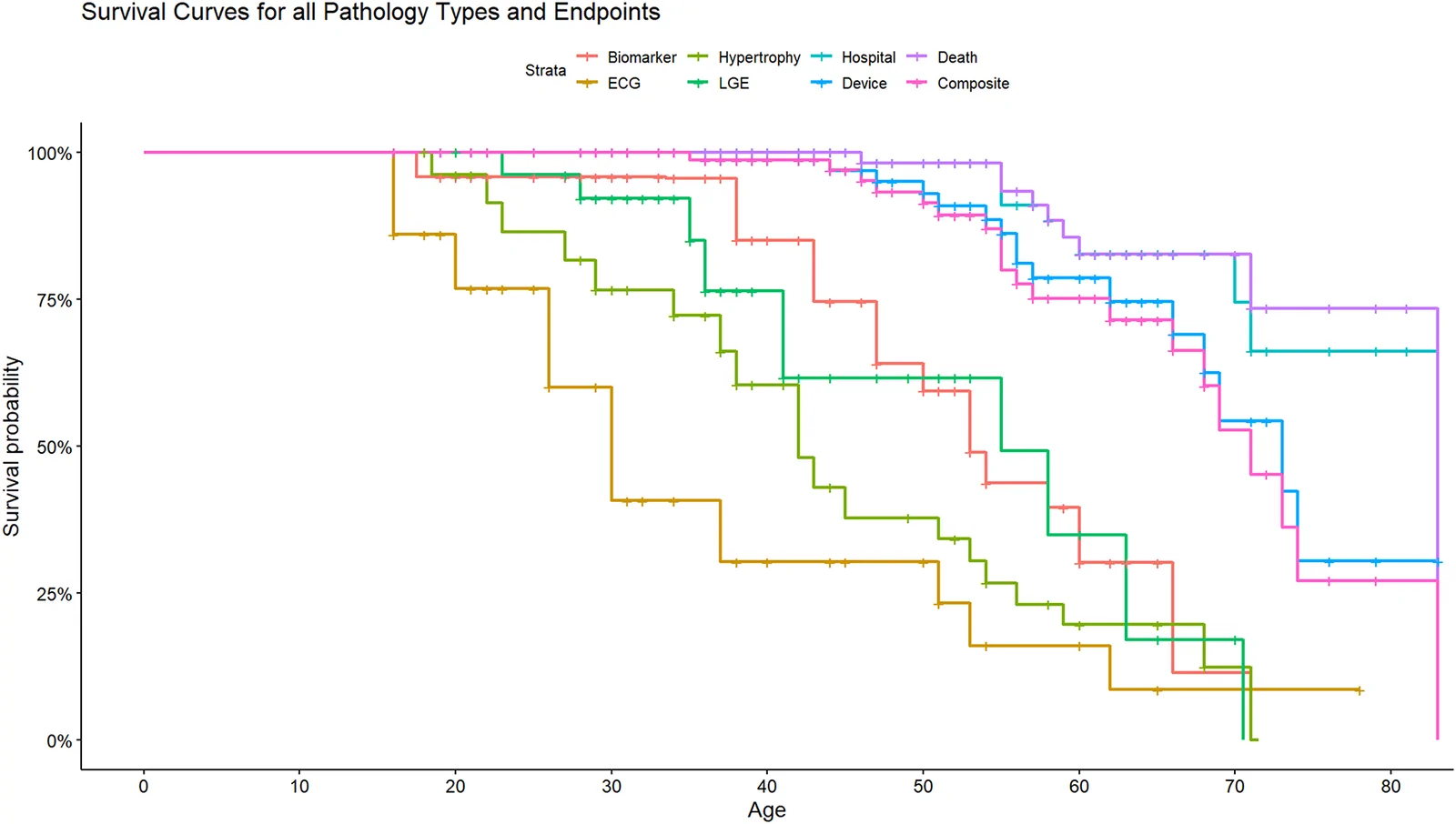
Survival curves for the incidence of CPC 1 for cardiac modalities and clinical outcomes

Finally, to determine the sequential development of FD cardiomyopathy according to all different CPC domains, a survival probability figure was plotted, summarizing the probability to progress from CPC 0 to CPC 1, as well as clinical endpoints (Fig. 4). This analysis suggests that transitions occur in a specific order, where ECG and imaging precede increase in biomarkers and presence of LGE.

In a supplementary analysis, we investigated whether LGE may precede hypertrophic changes specifically in females, as suggested by Niemann et al. [16]. In this sub-analysis, we focused on cases which reached CPC = 1 in both domains. There were 7 such cases: in 4 of them LGE onset preceded Hypertrophy onset, and in 3 Hypertrophy onset preceded LGE onset. In the latter 3 cases, Hypertrophy onset preceded LGE onset by 1 or 2 years.

## Discussion

In this study, we examined the combined effects of age, sex, mutation type, ECG findings, cardiac biomarkers, left ventricular hypertrophy (LVH), and late gadolinium enhancement (LGE) on cardiovascular outcomes in Fabry disease (FD). We also delineated the typical sequence of events in FD-related cardiac involvement. Our findings are consistent with data from the Fabry registry, reinforcing that males and those carrying classic mutations are at higher risk of cardiovascular events [17]. By developing and applying a four-domain CPC approach, we attempted to characterize the natural history of FD cardiomyopathy in a real-world setting over an extended period of follow-up.

A robust predictive model to identify adverse cardiac events in FD could transform clinical management by informing decisions about when to intensify monitoring or initiate interventions – for instance, considering device implantation or advanced imaging. Unlike a recent study that primarily assessed CMR findings to predict cardiac outcomes, our work incorporated clinical practice data, including scenarios where CMR was not always readily available [18]. This design enhances the generalizability of our approach to centers where CMR access is limited.

Our analysis shows that in line with prior literature, males with a classic GLA mutation reach abnormal CPC thresholds significantly earlier. Interestingly, both LGE and ECG emerged as potentially valuable predictors of future cardiac events, whereas cardiac biomarkers did not show strong predictive utility in this cohort. This finding underscores the diagnostic and prognostic complexities in FD, particularly given the frequent coexistence of renal impairment, which can confound biomarker interpretation. Moreover, it highlights the ongoing debate regarding the optimal timing and sequence of evaluations in FD: while annual assessments may be resource-intensive, delayed or infrequent follow-up could miss key junctures when patients transition from low-risk to high-risk states.

One of the noteworthy observations in our cohort was that for many individuals, ECG abnormalities arose years before any other cardiac findings. If validated in larger cohorts, this suggests a possible strategy wherein more frequent assessments might safely begin only after an ECG first becomes abnormal. When LGE subsequently appears, it may further stratify patients into a higher-risk group warranting close follow-up and earlier prophylactic interventions. However, we also acknowledge that an abnormal ECG could trigger device implantation in clinical practice, potentially confounding its predictive value in regression analyses. The 4-domain CPC model is intended as a systematic tracking tool that can inform surveillance intensity. Patients who transition to LGE CPC-1, identified here as the strongest independent predictor of composite adverse outcomes, represent a group warranting closer monitoring and earlier consideration of escalation of care. Conversely, patients remaining in early CPC stages — particularly those with late-onset mutations or female sex — may be followed at longer intervals, supporting a more individualised and resource-conscious approach to FD surveillance.

The complexity of FD in females is especially evident. Although phenotypic variability results from lyonization, our data suggest that genotype-based classification still helps delineate risk and disease trajectories in female patients. Females carrying a classic mutation showed more heterogeneous and, in some cases, earlier progression patterns compared to late-onset genotypes. The relative benign course in late-onset females, with very few reaching significant cardiac endpoints, further underscores the role of genotype in risk stratification. The clear separation between the outcomes of classic and late-onset females, as defined by genotype, lends support to this approach to classification, rather than a system based on current phenotype. Nevertheless, the variability introduced by X-inactivation remains a major challenge for uniform classification.

Several limitations warrant consideration. First, FD is an ultra-rare condition, and our sample size—though comparable to other studies—limits the statistical power to detect subtler trends [19, 20]. Additionally, the long study period introduced variability in data capture: older ECG reports were not always digitally archived, and laboratory assays for cardiac biomarkers changed over time. CMR use and protocols evolved substantially throughout the study, and parametric mapping was only recently implemented in our center, precluding its inclusion in this analysis. Finally, our population was limited to adult patients in a single tertiary referral center, leaving the earlier pediatric disease evolution under-represented.

Despite these constraints, the integration of diverse cardiac data from multiple evaluations—and the long duration of consistent follow-up—represents a strength, allowing us to characterize a potential timeline for FD cardiomyopathy. The four-domain CPC method offered a systematic approach to track progression within each of the included parameters. Our findings also illuminate referral patterns, particularly the tendency for males with late-onset FD to enter specialist care at older ages and in more advanced CPC. This might reflect emerging diagnostic pathways, including routine genetic panels for hypertrophic cardiomyopathy and increasing CMR use, both of which can detect FD in previously unsuspected cases.

Going forward, larger multicenter studies and machine-learning–based analyses could validate and refine the CPC framework, potentially leading to more nuanced and unified staging systems that capitalize on continuous rather than binary data. Moreover, expanding research into pediatric populations and exploring novel imaging techniques—such as parametric mapping—could further clarify the earliest stages of FD cardiomyopathy. Ultimately, our results highlight how synthesizing existing modalities into a cohesive predictive model can support a more patient-centered, potentially cost-effective approach to managing FD, and may also have relevance for other metabolic and infiltrative cardiomyopathies.

## Conclusions

The presented data endorse a “4 domain CPC” model as a strategic tool for monitoring FD cardiomyopathy evolution. Distinct trajectories determined by the combined effects of sex and mutation in FD were evident. The findings accentuate LGE, and to some degree ECG, as probable indicators for FD cardiomyopathy progression, while the definitive role of cardiac biomarkers remains to be elucidated.

## Electronic Supplementary Material

Below is the link to the electronic supplementary material.


Supplementary Material 1: Supplementary Figure 1: Counts of CPC1 to CPC0 (abnormal to normal) reversions across four cardiac phenotypic domains (ECG, Biomarkers, Hypertrophy, LGE), stratified by genotype/sex subgroups (All, Late-onset female/male, Classic female/male). Bars represent the number of patients with at least one observed reversion within each domain. In the aggregated cohort (All), reversions were infrequent (Biomarkers: 6; Hypertrophy: 8; ECG: 3; LGE: 0)


## Data Availability

The anonymized dataset, including cardiac phenotypic clusters can be made available upon email request to the corresponding author.
